# Effect of bottled fluoridated water to prevent dental caries in primary teeth: study protocol for a phase 2 parallel-group 3.5-year randomized controlled clinical trial (waterBEST)

**DOI:** 10.1186/s13063-024-08000-4

**Published:** 2024-03-05

**Authors:** Anne E. Sanders, Kimon Divaris, Tewodros R. Godebo, Gary D. Slade

**Affiliations:** 1 The University of North Carolina at Chapel Hill, Chapel Hill, NC USA; 2 Department of Environmental Health Sciences, Tulane University, New Orleans, LA USA

**Keywords:** Primary prevention, Community drinking water, Dental caries, Primary prevention, Randomized controlled trial

## Abstract

**Background:**

Fluoridation of public water systems is known as a safe and effective strategy for preventing dental caries based on evidence from non-randomized studies. Yet 110 million Americans do not have access to a fluoridated public water system and many others do not drink tap water. This article describes the study protocol for the first randomized controlled trial (RCT) of fluoridated water that assesses its potential dental caries preventive efficacy when delivered in bottles.

**Methods:**

waterBEST is a phase 2b proof-of-concept, randomized, quadruple-masked, placebo-controlled, parallel-group trial designed to estimate the potential efficacy of fluoridated versus non-fluoridated bottled water to prevent dental caries incidence in the first 4 years of life. Two hundred children living in eastern North Carolina, USA, and aged 2–6 months at screening are being allocated at random in a 1:1 ratio to receive fluoridated (0.7 mg/L F) or non-fluoridated bottled water sourced from two local public water systems. Throughout the 3.5-year intervention, study water is delivered monthly in 5-gallon bottles to each child’s home with instructions to use it whenever the child consumes water as a beverage or in food preparation. Parents are interviewed quarterly to monitor children’s water consumption and health. At annual visits, the presence of dental caries is evaluated with a dental screening examination. Clippings from fingernails and toenails are collected to quantify fluoride content as a biomarker of total fluoride intake. The primary endpoint is the number of primary tooth surfaces decayed, missing, or filled due to dental caries measured by the study dentist near the time of the child’s fourth birthday. Tooth decay is assessed at the threshold of macroscopic enamel loss. For the primary aim, a least-squares, generalized linear model will estimate efficacy and its one-tailed, upper 80% confidence limit.

**Discussion:**

waterBEST is the first evaluation of a randomized intervention of fluoridated drinking water in bottles to prevent dental caries in the primary dentition. This innovative method of delivering fluoridated water has the potential to prevent early childhood caries in a large segment of the US population that currently does not benefit from fluoridated public water.

**Trial registration:**

ClinicalTrials.gov NCT04893681. Registered on March 2022. Last update posted on 10 October 2023. https://clinicaltrials.gov/study/NCT04893681?cond=Dental%20Caries%20in%20Children&term=fluoride&locStr=North%20Carolina,%20USA&country=United%20States&state=North%20Carolina&distance=50&rank=1

## Introduction

### Background and rationale {6a}

Community water fluoridation is the controlled adjustment of fluoride in a public water system to a level that is optimal for preventing dental caries (i.e., tooth decay) while minimizing the risk of dental fluorosis (i.e., enamel hypomineralization). At the recommended concentration of 0.7 mg/L [1], fluoridated water reduces the prevalence and severity of dental caries in both primary and permanent dentitions. In fact, because water fluoridation reduces the excess fraction of dental caries in children in low-income relative to higher-income households [2], it also reduces income-related disparities in the disease.

A 2015 Cochrane review of the public health impact of community water fluoridation synthesized observational studies worldwide that compared a population receiving fluoridated water against a population receiving non-fluoridated water and that evaluated outcomes at least two points in time [3]. It found a caries prevented fraction of 35% in the primary dentition (i.e., deciduous teeth) and of 26% in the permanent dentition. While informative, 71% of the selected studies were conducted before 1975, prior to the widespread use of fluoride toothpaste. To address this gap and to narrow the focus to the US, we merged US county-level estimates of the percentage of the population served by community fluoridated water, obtained from the Centers for Disease Control and Prevention’s (CDC) Water Fluoridation Reporting System, with individual-level dental examination data from National Health and Nutrition Examination Surveys (1999 to 2004 and 2011 to 2014) [4]. Analysis compared dental caries experience in 19,604 children and adolescents who lived either in counties in which ≥ 75% of households were served by fluoridated community water systems or in counties with lower population coverage. We found that even with near-universal use of fluoride toothpaste, dental caries prevented fraction was 30% (95% CL = 11, 48) in the primary dentition, and 12% (95% CL = 1, 23) in the permanent dentition.

As well as being equitable and effective, community water fluoridation is cost-effective. Each year it saves the US an estimated $6.5 billion in averted direct and indirect treatment costs [5]. In fact, the combination of its low implementation costs coupled with its effectiveness at reducing dental caries prompted the Centers for Disease Control and Prevention to name water fluoridation among the ten great public health achievements of the twentieth century [6].

Approximately 200 million Americans, equivalent to 73% of the US population, are served by community water systems containing fluoride at the optimal level [7]. That leaves 110 million Americans, or one in four, unserved by fluoridated community water systems. Many of these water systems will remain unfluoridated because the rapid expansion in population coverage of fluoridated water that characterized the 1980s has now stalled. In fact, in the decade 2008–2018, population coverage increased by less than 1% from 72.4 to 73.0% [8]. Zokaie and Pollick [9] attribute the stalled expansion to the following: political hurdles to implementing a water fluoridation system, unfounded claims of adverse health effects of fluoridated water, and the view that fluoridation is no longer necessary as dental caries has ceased to be a public health problem. The latter is a misperception as a 2019 CDC report showed that dental caries affects the primary teeth of 23% of children aged 2–5 years and the permanent teeth of 37% of adolescents aged 12–19 years [10].

The American public’s growing distrust in the safety of tap water further limits exposure to fluoridated water. Over the period 2013–2018, avoidance of tap water in the US increased from 13 to 20% [11], consistent with greater consumption of bottled water [12]. Virtually all bottled water sold today is unfluoridated, which likely accounts for higher rates of dental caries observed in US children and adolescents who avoid drinking tap water compared to those who drink tap water [13].

So that more of these Americans can access fluoridated drinking water, the waterBEST study is investigating the delivery of fluoridated water in 5-gallon bottles to children living in eastern North Carolina. These children are at elevated risk of dental caries either because many of the water supplies in the region are not fluoridated or because they actively avoid drinking tap water.

Based on the SPIRIT checklist [14], this paper describes the protocol of the waterBEST trial https://waterbeststudy.com/ which is investigating if fluoride in bottled water helps to prevent dental caries in the first 4 years of life.

### Objectives {7}

The primary objective is to estimate the dental caries preventive efficacy of fluoridated bottled water consumption compared to non-fluoridated bottled water consumption in 4-year-olds. Efficacy is defined as the estimated mean difference in the number of decayed, missing, and filled tooth surfaces per child (dmfs) between intervention groups. Its one-sided, upper 80% confidence limit will be calculated to address inferences about plausible efficacy in a future phase 3 trial. The secondary objectives are to describe cohort retention and intervention adherence in each study group, compare safety parameters and total fluoride intake between study groups, and explore potential mediating effects of ingested fluoride from water on the efficacy of the intervention.

The secondary objectives are to:

- Assess intervention safety and key aspects of intervention adherence.
- Quantify study participation rate and cohort retention during follow-up.
- Determine the intervention’s effect on total fluoride intake.
- Estimate the contribution of ingested water to any preventive effect of fluoridated bottled water on dental caries.

### Trial design {8}

**Fig. 1:**
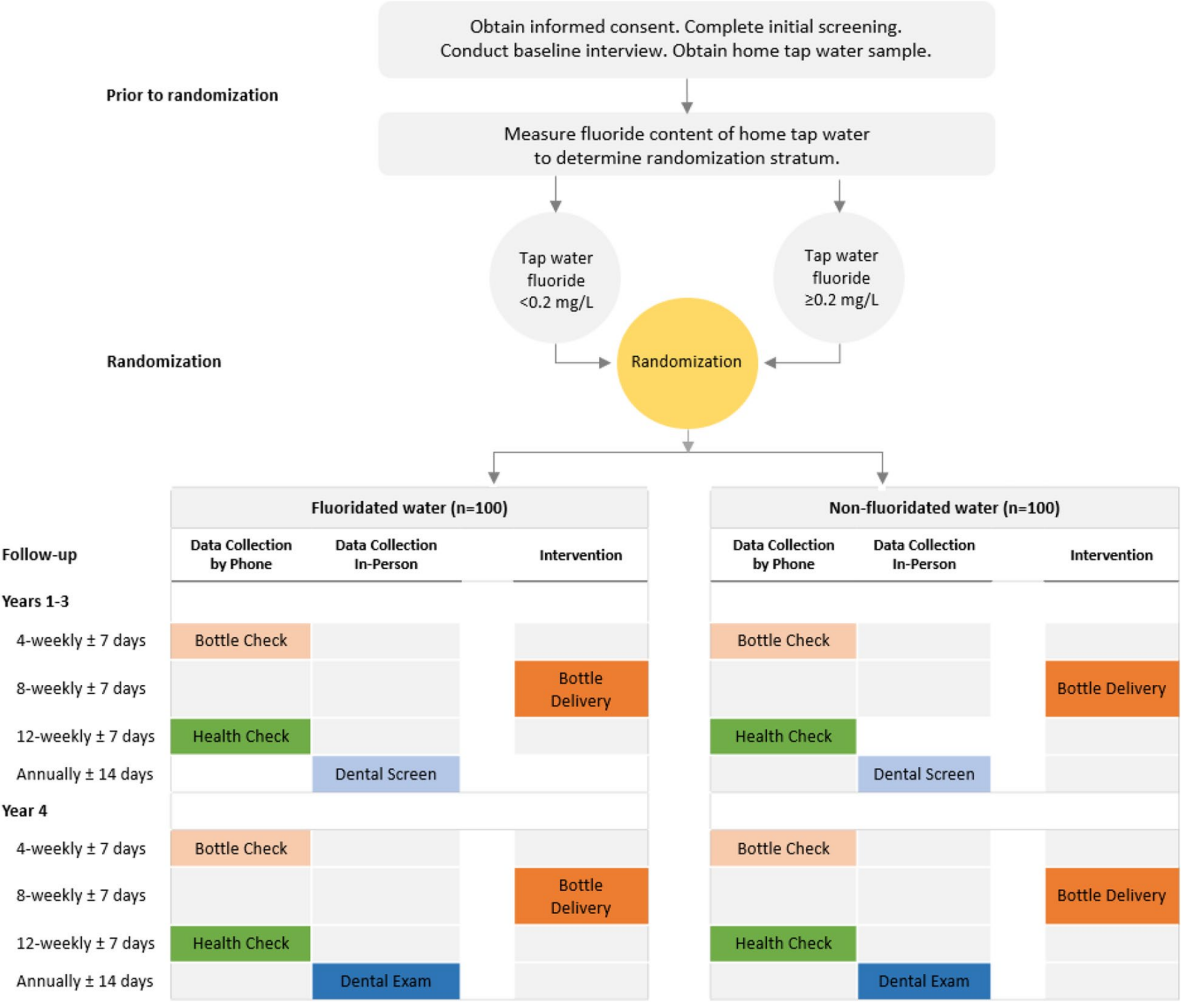
Schematic of the study design

The trial design is a phase 2b proof-of-concept, block randomized, quadruple-masked (participants, investigators, outcomes assessor), placebo-controlled, parallel-group trial evaluating dental caries-preventive effects of fluoridated bottled water compared to non-fluoridated bottled water in young children. Two hundred healthy children aged 2–6-months, along with their parent(s), guardian, or caregiver {hereafter referred to as “parent”}, will be recruited from Lenoir County, North Carolina (NC), or a neighboring county in eastern NC (Wayne, Pitt, Craven Counties). Children will be allocated at random in a 1:1 ratio to receive either fluoridated or non-fluoridated bottled water (Fig. 1).

Randomization will be done within each of two strata defined according to the fluoride content of tap water at the child’s primary dwelling: (a) <0.20 mg/L F or (b) ≥ 0.20 mg/L F. The duration of the intervention is 3.5 years.

**Fig. 2:**
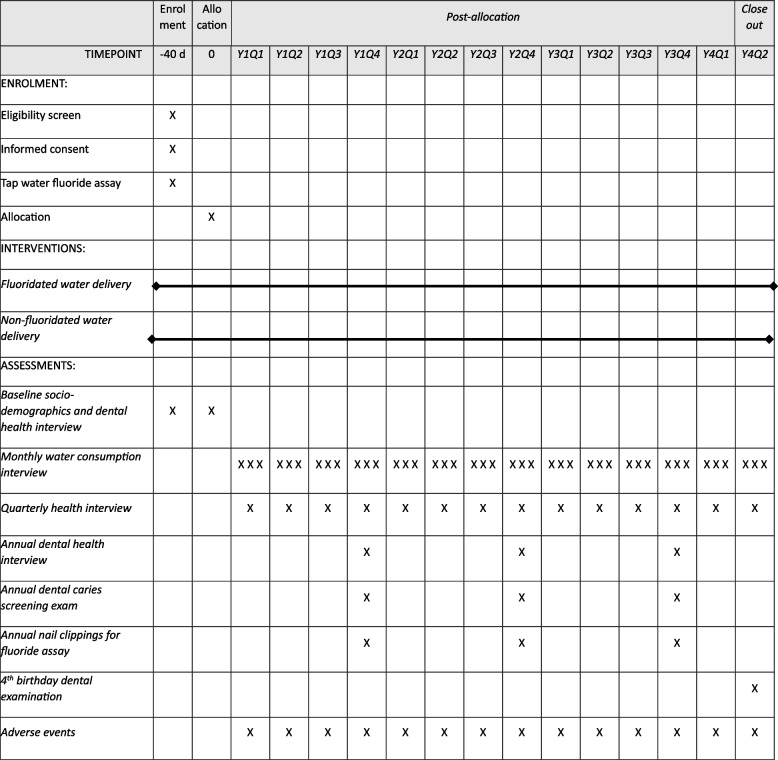
Schedule of enrolment, interventions, and assessments for the waterBEST study. This schedule depicts 42 encounters during the intervention period, representing a child who enrolls at 6 months of age and completes the study on or near their 4th birthday. Total number of encounters can vary from 38 to 48, varying according to age at enrollment and age at study completion

The schedule of enrolment, interventions, and assessments for the waterBEST study is depicted in Fig. 2.

### Methods: participants, interventions, and outcomes

#### Study setting {9}

The study is being conducted in Lenoir Co., NC, USA, and three neighboring eastern NC counties (Wayne, Pitt, Craven). Lenoir Co. was selected as the primary target population because it is the state’s largest city that does not fluoridate its public water system. The initial target for recruitment is children living in Kinston, the largest town and county seat. Kinston has a population exceeding 40,000 in which one third lives below the poverty level. The ~150 children born in the remainder of Lenoir Co. and in three neighboring counties are a secondary target population.

#### Eligibility criteria {10}

See the inclusion and exclusion criteria in Table 1.

**Table 1:** Inclusion and exclusion criteria

Inclusion criteria	Exclusion criteria
The child’s primary dwelling is in Lenoir County, NC (the primary target population) or one of three neighboring counties (Wayne, Craven, and Pitt).	Child has a serious illness/es requiring frequent inpatient hospitalization, as reported by the parent at the screening visit.
The child’s parent understands and consents to procedures described in the parental permission and consent form.	Child’s birth weight was less than 1500 g (3 pounds 5 ounces).
Child is aged 2 months to 6 months at the screening visit.	Child’s gestational age was less than 34 weeks.
Child either drinks water (either plain water or water mixed with something) or is expected to drink water by their first birthday, as reported by the parent at the screening visit.	Child uses fluoride supplements, as reported by the parent at the screening visit.
	The child’s primary dwelling at the enrollment visit has tap water that contains > 0.60 mg/L F and the parent/guardian/caregiver expresses a preference that the child drink tap water, not bottled water, for the child’s first 4 years of life. Fluoride concentration of a sample of the dwelling’s tap water is measured at Dr. Godebo’s laboratory using the Ion Selective Electrode method. For children who live at more than one dwelling, the primary dwelling is defined as the one at which they usually sleep at least four nights per week.
	Parent states at the screening visit the child will initiate fluoride supplementation before the child’s 4th birthday.
	The parent anticipates that, before the child’s 4th birthday, the child will move to an address more than 30 miles from their address at the time of enrollment.
	The investigators determine that a child living at the same primary dwelling has already been enrolled in the study. (This means that if two or more children fulfilling the inclusion criteria live at the primary dwelling, the parent will be asked to select one such child to be the study participant.)
	Anything that, in the opinion of the principal investigator, would place the participant at increased risk or preclude the participant’s full compliance with or completion of the study.

#### Who will obtain informed consent? {26a}

Trained study personnel obtain informed consent using the *Parental Permission for a Minor Child to Participate in a Research Study* Consent Form. Because children are not legally able to provide consent, federal regulations require that the parents provide permission before children can be enrolled in research. Furthermore, the UNC Institutional Review Board (IRB) has determined that the waterBEST study entails no more than minimal risk, and therefore, the permission of one parent is sufficient. The study personnel explain the research study to the child’s parent, including the annual collection of fingernail and toenail clippings, and answer any questions that may arise to the parent’s satisfaction. They provide sufficient time to decide whether to participate in the trial before obtaining consent. Study staff then ask questions to verify parents’ understanding of the study and record the answers to document the process of informed consent. Once the child’s parent understands and consents to procedures described in the parental permission and consent form, they sign the informed consent document prior to any study-related assessments or procedures.

*Additional consent provisions for collection and use of participant data and biological specimens {26b}*

This research is covered by a Certificate of Confidentiality, which is issued by the National Institutes of Health to safeguard the privacy of research study participants by protecting identifiable research information from forced disclosure.

### Interventions

#### Explanation for the choice of comparators {6b}

The source of the placebo is groundwater obtained from the Black Creek aquifer in Lenoir County, NC, by the North Lenoir Water Corporation, who treat it for public consumption consistent with state and federal requirements for public water systems. It contains a negligible concentration of approximately 0.3 mg/L fluoride.

#### Intervention description {11a}

The study products are drinking water bottled from two public water systems in eastern NC: the New Bern Water Resources Division’s Black Creek aquifer which contains naturally occurring fluoride in a concentration of approximately 0.8 mg/L F and the North Lenoir Water Corporation’s Black Creek aquifer which contains a negligible concentration of fluoride. Both public water systems produce drinking water consistent with state and federal requirements for public water systems [15, 16]. waterBEST study staff collect 600-gallon batches of potable water from each system and transport the batch to the Kinston field center. On the same day, the water is disinfected using ozone sterilization and packaged into 5-gallon bottles using an Ultra 150 bottling system. The Ultra 150 is a fully automated clean-room system that fulfills the requirements of the FDA and FSMA for processing drinking water intended for human consumption.

#### Criteria for discontinuing or modifying allocated interventions {11b}

The PI may discontinue an individual’s intervention if:

- The enrolled child begins taking fluoride supplements, as reported by the parent during quarterly health checks.
- The child moves to a primary dwelling where the concentration of fluoride in tap water exceeds 0.60 mg/L F and the parent has expressed a preference that the child drinks tap water.

The PI may withdraw an individual from the study if:

- A child relocates to a primary dwelling that is more than 30 miles from their address at the time of enrollment.
- Any adverse event or other medical condition or situation occurs such that continued participation in the study would not be in the best interest of the participant.
- The participant meets an exclusion criterion (either newly developed or not previously recognized) that precludes further study participation.

#### Strategies to improve adherence to interventions {11c}

Assessment of adherence with intended water use will be quantified using data about water consumption reported by the parent in monthly interviews throughout the 3.5 years of follow-up. In addition, the number of bottles delivered to each dwelling is recorded, along with the number of people at the dwelling who consume study water. Rates of delivery of study water, consumption of study water, and consumption of non-study water during follow-up will each be assessed as measures of intervention adherence.

To promote adherence to the interventions, each dwelling is given a bottom-loading water dispenser that dispenses hot, cold, and room-temperature water at the push of a button. Study staff demonstrate how to load bottles, dispense water, and replace empty bottles as needed, and they give written instructions. Age-appropriate cups, spill-proof bottles, and related drinking aids will also be provided at no cost to study participants. A travel bag is provided to store water and drinking supplies for use when children are away from home.

#### Relevant concomitant care permitted or prohibited during the trial {11d}

All parents are encouraged to clean their child’s teeth using toothpaste. To avoid the risk of ingesting too much toothpaste (e.g., accidentally swallowing half a tube of toothpaste), the parent is given information about fluoride intake, including correct use of toothpaste. Regular dental visits are permitted.

The use of fluoride supplements and use of study water to reconstitute infant formula are not permitted because of the risk of excessive fluoride intake and subsequent dental fluorosis. At quarterly health checks, study staff interview parents and ask questions about the child’s use of fluoride supplements and infant formula. If the child begins taking a fluoride supplement, the intervention is discontinued for the duration of supplement usage. For study children being fed infant formula concentrate, waterBEST provides 3-gallon bottles of non-fluoridated water purchased from Le Bleu Central Distributors of Wilson, NC, with instructions that it be used solely for reconstitution of formula.

#### Provisions for post-trial care {30}

Parents are given a written report of their child’s dental health after the annual dental screening assessment and after the end-of-study dental caries examination. This will include recommendations for dental care, if required.

##### Outcomes {12}

The primary outcome for efficacy evaluation is the dmfs index, a measure of the dental caries experience in the study participant’s primary dentition. It is quantified as the number of primary tooth surfaces (s) that are decayed (d), missing (m), or filled (f). This index is enumerated during a dental examination near the time of the child’s 4th birthday. Efficacy is defined as the estimated difference in adjusted mean dmfs per child between intervention groups, calculated using a least squares general linear model that adjusts for randomization stratum and age in months at the time of the examination. The index of decayed, missing, and filled primary tooth surfaces (dmfs) is the most widely used, validated, and clinically meaningful epidemiological endpoint of dental caries experience in the primary dentition of children in this age range.

There are four secondary outcomes:

Intervention safety is being determined at quarterly health checks by asking parent/guardian about potential adverse events. To determine if differences exist between study groups in rates of adverse events, rates for each study group will be computed as the total number of adverse events (numerator) divided by person-time of follow-up (denominator).

Study participation and cohort retention during follow-up will be enumerated as the number of participants screened, randomized, and completing follow-up data collection. Outcomes will be as follows: proportion of screened participants providing informed consent, proportion of consenting participants randomized, proportion of randomized participants completing follow-up data collection.

Intervention adherence is being determined with questions to parents/guardians at monthly Bottle Consumption Visits about consumption of study water, and consumption of non-study water during follow-up. Intervention adherence will be computed as the usual number of times per day that study water is consumed, either as plain water or when mixed with something. Monthly reports for each study participant will be integrated to create a subject-specific density measure of water consumption by plotting each month’s reported frequency on the vertical axis and the child’s age (in months) on the horizontal axis of a time-series plot for the study participant. The area under the curve of the time series will be calculated using the trapezoid method to represent the study participant’s frequency measure of intervention adherence.

The intervention’s effect on total fluoride intake will be determined from the fluoride content of fingernail and toenail biospecimens, a biomarker of total fluoride intake. Clippings for fingernails and toenails are collected at intervals of 1, 2, and 3 years after randomization. The fluoride content of fingernail and toenail clippings is being measured in mg/kg using a hexamethyldisiloxane-facilitated diffusion assay of nail clippings collected from study participants. The intervention’s impact on total fluoride consumption will be estimated using measurements of mean fluoride content of fingernail biospecimens.

##### Participant timeline {13}

See Figure 3.

**Fig. 3:**
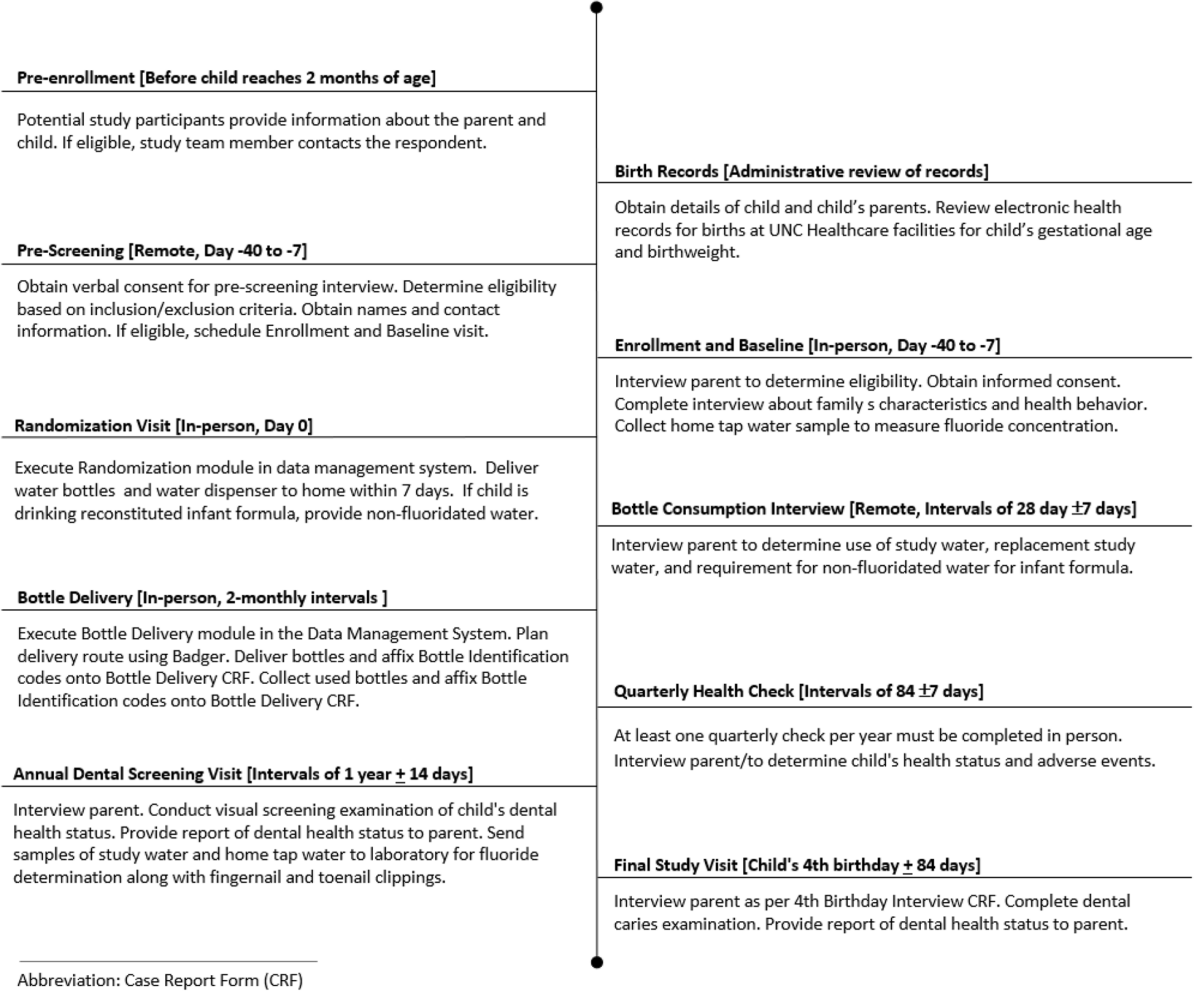
Participant timeline

##### Sample size {14}

The power calculation for this phase 2b trial is based on the principle that pilot studies should replicate, in miniature, a definitive trial that would be conducted if justified by findings from the pilot study [17]. By definition, such pilot studies lack the statistical power of a definitive trial. Instead, the goal is to calculate the point estimate of the preventive effect size and plausible range of effect size estimates to make the following three decisions:

- If the pilot study’s point estimate of effect size is zero or less, and there were no significant flaws in the conduct of the pilot study, a definitive trial is contra-indicated (i.e., a null or harmful finding).
- If the one-tailed, upper 80% confidence limit of the pilot study effect size is less than the a priori threshold of public-health-significance, then a definitive trial is probably unjustifiable because it would require a very large sample size to estimate a meager health benefit.
- Otherwise, the definitive trial should go ahead using the point estimate from the pilot study as the effect size when calculating the required sample size for the definitive trial.

When applied using estimates of the dmfs index for 3–4 year-olds living in non-fluoridated areas of the US [4], that approach yields a required sample size of 158 subjects (79 per study group), specifying a clinically significant group difference of 1.1 (s.d. = 7.8), equivalent to 37% reduction in mean dmfs, and power of 80%. (Specifically, using the SAS power procedure with options “two sample means ci = diff halfwidth = 1.1, stddev = 7.8,” 1-tailed alpha = 0.2 and probwidth = 0.8.) The required sample size of 158 subjects with 4-year-old dmfs data was increased to 200 randomized subjects to allow for a presumed loss to follow-up of 20% between enrollment and the 4th Birthday Dental Caries Examination.

##### Recruitment {15}

To randomize 200 children, the primary recruitment strategy is a direct approach by study personnel to families of children whose births are recorded in birth certificates which are freely available as a public record at Register of Deeds offices at in all four counties. By law, all births in the county must be registered, assuring that our recruitment efforts will identify children from all sex/gender, and racial and ethnic backgrounds. Details from the child’s birth records, including parents’ names and the child’s name and address, are transcribed by study personnel. When the child is aged 2 months, study staff mail a letter to the child’s home address and follow up with door-knock visits. The recruitment strategy is designed to achieve a distribution of demographic characteristics in the study sample mirroring that of the target population of infants in Lenoir County, NC.

The secondary strategy is a pre-enrollment questionnaire that collects contact information of people who express interest in the study prior to their child reaching the enrollment-eligibility age of 2 months. Interested individuals can record their contact information during an interview with study representatives or by completing a questionnaire on paper or online. Traditional recruitment strategies include word of mouth; recruitment flyers placed in health care facilities, community, and welfare organizations; and social media accounts.

### Assignment of interventions: allocation

#### Sequence generation {16a}

Randomization is stratified according to fluoride concentration in the tap water of the child’s primary dwelling:

•< 0.20 mg/L F inclusive of levels below the threshold of measurement

•≥ 0.20 mg/L F

Within each stratum, and within pre-specified blocks, participants are randomized in a 1:1 ratio to either the fluoridated or non-fluoridated study group. The allocation sequence and study group assignment are generated using the “plan” procedure in SAS v9.4 software.

#### Concealment mechanism {16b}

The allocation sequence is stored in a “split-half” segment of the study database that is protected by a password known only to the unmasked data manager and (as backup) the study biostatistician, thereby concealing the allocation sequence to users. Neither the unmasked data manager nor the study biostatistician is involved in the process of randomizing individuals.

#### Implementation {16c}

Study staff implement the allocation sequence using a randomization module of the Data Management System (DMS). After entering data to verify the study participant’s eligibility and stratum for randomization, staff click a button that executes SQL code which allocates the study participant to one study group, and then assigns the record number of the next, unused row of the allocation table along with the study participant’s 3-digit identification code. The DMS generates a verification code that is stored in the randomization table and recorded in the participant’s casebook providing an audit trail to document that the study participant was randomized. In this way, the DMS does not reveal the study participant’s study group assignment nor the allocation sequence to users.

### Assignment of interventions: blinding

#### Who will be blinded {17a}

The study has quadruple masking. Participants, field center staff, investigators, and clinical examiners are masked as to the participants’ study group assignment throughout the data collection period. Only the unmasked data manager (and, as backup, the study statistician) is not masked.

In order to mask participant’s study group, study personnel and participants are made unaware of the contents of 5-gallon bottles of water delivered to dwellings. This is achieved by a masking procedure that assigns a unique, 5-digit serial number at random to each 5-gallon bottle of water after it has been filled from either the New Bern (fluoridated) or North Lenoir (non-fluoridated) public water systems. Masking is achieved in three steps, each performed by a separate study staff person, and each masked bottle is placed at random into a labeled position in racks that can store 768 bottles. Masked bottles from any single public water system are therefore distributed at random among rack positions and the bottles are identifiable to staff only by their 5-digit identifying code.

When new water bottles are requested at monthly Bottle Consumption interviews, staff execute a bottle allocation routine in the DMS. The underlying SAS code retrieves the study participant’s study group assignment and selects from the supply of masked bottles the necessary number of masked bottles that conform with the study participant’s treatment group assignment. Field center staff deliver then the water to the household.

#### Procedure for unblinding if needed {17b}

There are no anticipated circumstances in which an individual study participant will be unmasked. However, the unmasked data manager will generate the report if an oversight board, e.g., the IRB or Data and Safety Monitoring Board (DSMB), requests an unmasked data report during the data collection period.

### Data collection and management

#### Plans for assessment and collection of outcomes {18a}

Dental caries experience will be recorded on five surfaces of each primary tooth, with caries lesions defined at the threshold consistent with macroscopic enamel loss. Five surfaces per tooth will be enumerated in all primary teeth (maximum = 20 teeth per child), yielding a count of affected surfaces that can range from 0 (no dental caries experience) to 100 (worst possible extent of dental caries experience). The examination uses criteria of the International Caries Detection and Assessment System (ICDAS ≥ 3) [18]. A portable dental chair with overhead lighting and compressed air will be used in the home or comparable setting. After a toothbrush prophylaxis, children will be examined in a supine position with the aid of visual magnification. A research assistant will record data using dental charting software.

Training and calibration procedures for dental examinations will be led by Dr. Divaris (DDS) who is a board-certified pediatric dentist with extensive experience in the conduct of clinical examinations of preschool-age children for dental caries. Dr. Divaris will work with the study examiner, Dr. Ginnis, to review the online training modules available on the ICDAS website. This will be followed by a 2-h session with Dr. Divaris to review and discuss clinical cases. Clinical training and calibration sessions will take place during a 2-day session at UNC-Chapel Hill. Drs. Ginnis and Divaris will independently examine 20 preschool children ages 3–4 (who are not in the waterBEST study). Findings will be used to calculate inter-examiner agreement statistics (kappa, weighted kappa, percent agreement, and intraclass correlation coefficient) to quantify the examiner’s performance. An inter-examiner kappa score of ≥ 0.65 for binary classification of caries (IDCAS ≥ 3) will indicate that the examiner is calibrated. A repeat training will be held early in year 6, after one half of the examinations have been completed.

#### Plans to promote participant retention and complete follow-up {18b}

Study staff maintain close contact with families. A member of the study team calls families each month to assess their bottled water needs usage and visits their homes alternate months to deliver water bottles. These contacts create opportunities to keep track of changes in household schedule, changes of address, or disruptions to family routine.

When families move permanently out of the county, we will ask for contact information to keep in touch and potentially to conduct a dental examination near the time of the child’s fourth birthday. For such families, and for children whose intervention is discontinued prior to them completing the study, the parent is asked to give permission for the study team to contact the family near the time of the child’s fourth birthday to complete the 4th Birthday Interview.

Study participants are considered lost to follow-up when they cannot be contacted by the study team for the purpose of water bottle delivery and/or interviews or study visits. To minimize such loss, study participants are asked at enrollment to provide names and contact information for up to four third parties who would be willing to speak with the study team about the family’s whereabouts, in the event that the study team has trouble contacting enrolled study participants.

There is no replacement for study participants who are lost to follow-up.

#### Data management {19}

Study staff complete paper clinical report forms (CRFs) designed using Teleform software that is also used for scanning and optical recognition of responses. Electronic clinical report forms (eCRFs) are designed using Microsoft Access. The data are stored on an IRB-approved network drive at UNC-CH that is password protected with two-factor authentication required for remote access. CRF-level input edits enforce data entry for mandatory fields and use logic algorithms to implement skip sequences and to verify user decisions (for example, when determining eligibility based on responses to multiple variables). Data quality is ensured through the data collection system’s continuous monitoring of data and real-time detection and correction of errors. All elements of data entry (i.e., time, date, verbatim text, and the name of the person performing the data entry) are recorded in an electronic audit trail to allow all changes in the database to be monitored and maintained in accordance with applicable regulations.

Data collection and accurate documentation are the responsibility of the study staff under the supervision of the PI. All source documents and laboratory reports will be reviewed by the study team and data entry staff, who will ensure that they are accurate and complete. Unanticipated problems and adverse events must be reviewed by the PI or a designee.

The DMS generates study progress reports (i.e., screening, enrollment, cohort retention, and study progress reports) to the study website periodically throughout the study period, as determined by the NIDCR program official and the PI. The study team will provide monthly enrollment/retention reports to NIDCR for review. The DMS also generates management reports that contain measures of data quality, such as the number of outstanding data queries and data completion rates. Study reports are provided to the DSMB and IRBs prior to their periodic meetings. No interim statistical analyses are planned.

The DMS manages a relational database designed using Microsoft Access which will interact with SAS, Teleform, and Badger software. The main elements are summarized below.

- Information about potential participants is transcribed from birth records and entered into paper case report forms (pCRFs) which will then be scanned, encoded, and stored as a read-only electronic CRF (eCRF) in the DMS. As a quality control measure, each eCRF record will also store a read-only scanned image of the pCRF that was used to create the eCRF record. The term “read-only” means that users cannot change the stored data using the DMS.
- Any changes that must be made to CRFs (e.g., following the discovery of errors during QC procedures) will be recorded on the original pCRF (i.e., the source document). Changes will be annotated with a staff person’s initials and date, thereby providing an audit trail of changes. The revised pCRF will then be scanned and encoded using TeleForm and uploaded to the Data Management System which will keep a parallel system of audit trail in the eCRF.
- Other information about the target population, dwellings, and screening eligibility will likewise be recorded onto pCRFs and read-only eCRF copies will be stored in the DMS.
- Information about batches of bulk water purchased from public water utilities will be recorded using a pCRF. When the water is bottled the unmasked bottles will be labeled to signify the batch and one sample from each batch will be analyzed for water quality at the NC Division of Public Health, State Laboratory of Public Health.
- Bottle masking and storage will be managed by a SAS program called Microsoft Access, with records being written to Microsoft Access by the SAS SQL procedure.
- The 5-gallon bottles of interventional products will be distributed in a three-step process:
- Information about water consumption will be recorded using a pCRF and stored as an eCRF in the DMS.
- At weekly intervals, those records will be passed to a SAS program that will allocate masked bottles to the appropriate study participants, based on their study group assignment. The SAS program will then write records for the week’s delivery schedule to Microsoft Access, including an Access table that will initiate an Automerge function in Teleform, producing pre-filled pCRFs for each dwelling in the forthcoming delivery schedule.
- During each delivery route, records of delivered, full bottles and collected, empty bottles will be recorded onto the Water Bottle Delivery pCRF using bar-coded bottle identification labels. The barcode will also be scanned into the Badger mobile app, a route-planning program that uses GPS technology for geolocation during delivery. It also records whether or not the user is within a 200-m radius of the GPS coordinates of the intended address at the time of delivery.
- Data collected during study visits, including information about intervention adherence, safety, and efficacy, will be collected using pCRFs.
- Tap water, study water, and nail clippings shipments will be tracked to Dr. Godebo’s laboratory and data will be entered using eCRFs designed with Microsoft Access.

#### Confidentiality {27}

Personal information about potential and enrolled participants is strictly held in trust by the investigators, study staff, and the study sponsor(s) and their agents. This confidentiality is extended to cover the testing of biological samples in addition to any study information relating to participants. No information concerning the study or the data will be released to any unauthorized third party without prior written approval of the study sponsor.

The study monitor or other authorized representatives of NIDCR may inspect all study documents and records required to be maintained by the investigator, including but not limited to, medical records (office, clinic, or hospital) for the study participants. The clinical study site will permit access to such records.

#### *Plans for collection, laboratory evaluation, and storage of biological specimens for genetic or molecular analysis in this trial/future use {33***}**

Laboratory processes involved in measuring fluoride in fingernail and toenail clippings destroy the original material, rendering the nail clippings unable to be used for identification.

### Statistical methods

#### Statistical methods for primary and secondary outcomes {20a}

The statistical analysis for the primary objective will estimate efficacy and the one-tailed, upper 80% confidence limit of the estimate. Efficacy is defined as the estimated difference in adjusted mean dmfs per child between intervention groups, calculated with a general linear model estimated by ordinary least squares using the SAS “GLM” procedure. The dependent variable will be the child’s dmfs index, an integer (count) measure with a potential range of 0–100 surfaces. The main predictor variable will be the study group (modeled as a binary, dummy variable) and covariates will be randomization stratum (modeled as a binary, dummy variable) and child’s age, in months, at the time of the examination (modeled as a continuous variable). An “estimate” statement in the GLM procedure will calculate the adjusted mean and 1-tailed, upper 80% confidence limit.

#### Interim analyses {21b}

No interim analysis is planned.

#### Methods for additional analyses (e.g., subgroup analyses) {20b}

Potential variation in efficacy according to the degree of intervention compliance will be further explored within the per-protocol population. Within that population, tertiles of the frequency measure of intervention adherence will be calculated to create three strata of intervention adherence: low, moderate, and high. The stratum variable will be added as a class variable to the general linear model described above for the primary efficacy analysis, along with a treatment group × stratum interaction. Estimate statements in the SAS “GLM” procedure will be used to generate stratum-specific point estimates of the adjusted mean treatment effect and its 1-tailed, upper 80% confidence limit.

Exploratory analysis will estimate efficacy separately for boys and girls to investigate sex as a biological variable. Sex (male or female) will be added as a class variable to the general linear model described above for the primary efficacy analysis, along with a treatment group × sex interaction term. Estimate statements in the SAS “GLM” procedure will be used to generate sex-specific point estimates of the adjusted mean treatment effect and its 1-tailed, upper 80% confidence limit.

#### Methods in analysis to handle protocol non-adherence and any statistical methods to handle missing data {20c}

Multiple imputation of the missing endpoint will be investigated using information collected at annual dental screening and quarterly health updates. The analysis will be based on the strategy and principles described by Jakobsen et. al. [17] for multiple imputation as an exploratory analysis, including best-worst and worst-best sensitivity analyses. All findings will be reported to guide the interpretation of the primary analysis.

#### *Plans to give access to the full protocol, participant-level data, and statistical code {31c***}**

The full study protocol is available for download from the waterBEST study website (https://waterbeststudy.com/). Data arising from the study, including participant-level data, will be made available to the public as stipulated by NIH policy (https://sharing.nih.gov/data-management-and-sharing-policy/about-data-management-and-sharing-policies).

### Oversight and monitoring

#### Composition of the coordinating center and trial steering committee {5d}

Support for the trial’s operations will be provided by leaders of the investigative team and designated staff. The principal investigator, Slade, will provide overall direction for the trial’s scientific and administrative conduct. Slade will review day-to-day operations with the project manager and the two of them will meet weekly with field center staff whose duties include enrollment and informed consent. The principal investigator will also meet weekly with the following: (a) co-investigator Sanders who will direct staff engaged in study publicity which includes social media advertising for recruitment of study participants and (b) the study’s data manager who will manage data systems and electronic case report forms. Slade and Sanders will convene monthly meetings with NIDCR program officials who monitor the study’s progress. All investigators will meet annually with the study’s Data and Safety Monitoring Board (DSMB) to report on study participant safety, data quality, and overall progress of the trial. The study team will also draw on advice provided by the study’s Community Advisory Board that met four times during the study’s planning phase and which will meet annually after enrollment begins.

#### Composition of the data monitoring committee, its role, and reporting structure {21a}

Study oversight will be under the direction of a DSMB established by NIDCR which appointed members with appropriate expertise in clinical, statistical, scientific, and ethical areas. The DSMB will assess the study’s safety and efficacy data, progress, and data integrity. If safety concerns arise, more frequent meetings may be held. The DSMB will operate under the rules of an NIDCR-approved charter that will be approved at the organizational meeting of the DSMB. At this time, most data elements that the DSMB needs to assess will be clearly defined. The DSMB will provide recommendations to the NIDCR.

#### Adverse event reporting and harms {22}

During the quarterly interview, study staff will inquire about the child’s health status, health care, and adverse events (AE) associated with participation in the study. Study staff will record AEs using the Quarterly Health Check CRF administered during interviews conducted at 12-week intervals between enrollment and the 4th Birthday Dental Caries Examination. Because common childhood illnesses are expected to occur frequently during children’s first 4 years of life, and because it is implausible that such AEs could be attributed to the study products, common childhood illnesses will *not be reported as AEs* to the IRB or NIDCR. The following are examples of common childhood illnesses that will not be reported as AEs to the IRB or NIDCR:

- Infants and toddlers aged 0–3 years: common cold; ear infection; group B strep; influenza (flu); pertussis (whooping cough); recreational water illness (RWI); sinus infection; chickenpox; conjunctivitis (pink eye); diphtheria; foodborne diseases; hand, foot and mouth disease; jaundice (kernicterus); rotavirus; sore throat. (Source: https://www.cdc.gov/parents/infants/diseases_conditions.html)
- Children aged 4–11 years: attention-deficit/hyperactivity disorder (ADHD); asthma; chickenpox; coronavirus disease 2019 (COVID-19); influenza (flu); obesity. (Source: https://www.cdc.gov/parents/children/index.html)

The PI will monitor these events to grade severity and relationship to the study intervention and assess whether the nature, severity, or frequency are unexpected.

When participants withdraw from the study or their participation is terminated by the PI, the circumstances will be recorded in the Early Termination CRF and study staff will complete a study disposition form in the Data Management System. Any participant with an adverse event that is ongoing at the time of discontinuation will be followed until the event returns to baseline, resolves, or stabilizes.

#### Frequency and plans for auditing trial conduct {23}

Clinical site monitoring is conducted to ensure that the rights of human subjects are protected, that the study is implemented in accordance with the protocol and/or other operating procedures, and that the quality and integrity of study data and data collection methods are maintained. Monitoring for this study will be performed by NIDCR’s Clinical Research Operations and Management Support (CROMS) contractor, Rho. The monitor will evaluate study processes and documentation based on the International Council for Harmonization (ICH), E6: Good Clinical Practice guidelines (GCP).

Details of clinical site monitoring are documented in a Clinical Monitoring Plan (CMP). The CMP specifies the frequency of monitoring, monitoring procedures, the level of clinical site monitoring activities (e.g., the percentage of participant data to be reviewed), and the distribution of monitoring reports. Some monitoring activities may be performed remotely, while others will take place at the study site(s). Staff from CROMS will conduct monitoring activities and provide reports of the findings and associated action items in accordance with the details described in the CMP. Documentation of monitoring activities and findings will be provided to the site study team, the study PI, NIDCR-OCTOM, and NIDCR Program staff. The NIDCR reserves the right to conduct independent clinical site monitoring as necessary.

The NIDCR Medical Monitor provides independent safety monitoring. This is achieved by the evaluation of safety data with follow-up through resolution or stabilization, evaluation of unanticipated problems and protocol deviations, and review of data on disposition of biospecimens, outcome measures, and other relevant parameters.

#### Plans for communicating important protocol amendments to relevant parties (e.g., trial participants, ethical committees) {25}

All protocol changes are approved by the UNC Chapel Hill IRB and numbered versions of the protocol are provided to CROMS.

#### Dissemination plans {31a}

The results of the trial will be submitted for publication in peer-reviewed scientific journals within 12 months of the close of the study. Communities involved in the study will be informed of the outcomes and other stakeholders will receive relevant information.

## Discussion

The waterBEST study is the first RCT to test the dental caries preventive effects of fluoride in drinking water. Until now, the prevailing view was that fluoridated water could not be rigorously tested because fluoridation was limited to public water systems, and it was therefore impossible to randomly allocate individuals to the intervention or mask their allocation. That assumption no longer holds because bottled water offers an alternative modality for delivering masked water to households. The US is the largest consumer market of bottled water worldwide, with per capita consumption in 2022 reaching approximately 46.5 gallons [19]. Although most bottled water has fluoride concentrations well below the optimal 0.7 mg/L optimal for preventing dental caries, the controlled addition of fluoride is entirely feasible. Ironically, 25% of bottled water is currently obtained from municipal water supplies [20], yet many brands undergo reverse osmosis treatment which removes fluoride from the water.

A long-standing criticism raised by water fluoridation opponents is that there has never been a randomized controlled trial to demonstrate fluoridation’s effectiveness or safety [21]. Instead, the best evidence of the protective dental effects of water fluoridation comes from studies using a serial cross-sectional design. In 1944–1945, the US and Canada began paired community comparison studies in which four test cities raised the fluoridation concentration of public water supplies to 1.0 or 1.2 ppm, while a nearby paired control city of similar size and demographic composition retained its negligible fluoride concentration. These test and control cities respectively were Grand Rapids and Muskegon, Michigan; Newburgh and Kingston, New York; Evanston and Oak Park, Illinois; and Brantford and Sarnia, Ontario.

At the end of the observation periods, the longest of which was 15 years, the prevalence and severity of dental caries had sharply reduced in primary and permanent dentitions of participating school children in each test city. The overall findings were compelling, but methodological differences introduced bias and unnecessary random error [22]. For instance, different children were sampled each year to maintain selected age groups, sampling methods varied, dental examiners were replaced, and Muskegon (a control city) started fluoridation in 1951 [23]. Nonetheless, the rate of dental caries in the primary teeth of 6-year-olds reduced by about 54% on average, and the rate of dental caries in the permanent teeth of children aged 12–14 years reduced by 60% on average [24].

### Trial status

The current protocol version number is 1.4 dated November 11, 2022. Recruitment began in April 2022 and is expected to finish in September 2024.

## Data Availability

The trial data will be made available after the primary publication or 12 months after trial completion (whichever is earlier) upon reasonable request and in agreement with the Data Sharing Policy.

## Abbreviations

AE: Adverse events
ASTDD: Association of State and Territorial Dental Directors
dmfs: Sum of primary tooth surfaces that are decayed, missing and filled due to dental caries
CMP: Clinical Monitoring Plan
CROMS: Clinical Research Operations and Management Support
CRF: Case report form
CDC: Centers for Disease Control and Prevention
DMS: Data Management System
DSMB: Data Safety and Monitoring Board
ICH: International Council for Harmonization
GCP: Good Clinical Practice guidelines
IRB: Institutional Review Board
NC: North Carolina
NIDCR: National Institute of Dental and Craniofacial Research
NIH: National Institutes of Health
NHANES: National Health and Nutrition Examination Surveys
PI: Principal investigator
UNC-CH: University of North Carolina at Chapel Hill
waterBEST: Water from Bottles to Establish Strong Teeth
